# The effectiveness of an electronic pain management programme for the working population with chronic pain: study protocol for a randomized controlled trial

**DOI:** 10.1186/s13063-020-04348-5

**Published:** 2020-05-24

**Authors:** Shuk Kwan Tang, Mimi Mun Yee Tse, Sau Fong Leung, Theofanis Fotis

**Affiliations:** 1 School of Nursing, The Hong Kong Polytechnic University, Kowloon, Hong Kong; 2 School of Health Sciences, University of Brighton, Westlain House, Village Way, Brighton, BN1 9PH UK

**Keywords:** Pain management, Chronic pain, Working population, Electronic pain management programme, ePain, eHealth

## Abstract

**Background:**

Chronic pain is highly prevalent in the working population. People tend to attempt self-initiated treatments to manage their pain. The self-efficacy of behavioural change is a suitable model for guiding the development of an electronic pain management programme (ePain). The aim in this study is to develop ePain and to evaluate its effectiveness at improving pain self-efficacy, reducing pain intensity and negative emotions, and increasing quality of life.

**Methods:**

This study will be a randomized controlled trial. ePain will take the form of a 6-week online pain management programme. Participants will be aged 15 years or above, have chronic pain, and be employed. They must complete the baseline questionnaire and will be randomized into intervention and control groups. They will receive notifications to encourage their participation in ePain and complete the evaluation questionnaires. They will complete the process evaluation at week 3, the post assessment at week 6, and the follow-up assessment at week 12. The study will focus on pain self-efficacy; pain situations; negative emotions including levels of depression, anxiety, and stress; and quality of life. The participants’ opinions of ePain will be collected as feedback. Data will be analysed on an intention-to-treat basis and generalized estimating equations will be used to investigate the time-averaged difference and differences at each follow-up time.

**Discussion:**

The study will provide information about the pain situations of online users in the working population. The participants will benefit from improvements in pain self-efficacy, pain situations, emotional status, and quality of life. The study will illustrate whether online learning is an effective intervention for improving the pain self-efficacy of the working population.

**Trial registration:**

ClinicalTrials.gov, NCT03718702. Registered on 23 October 2018.

## Background

The working population comprises the largest portion of people in Hong Kong society and is defined as adults aged from 15 to 64 years [1]. This group of people helps society to function. However, some of them continue to work despite experiencing pain.

Pain affects all age groups. It is defined as ‘an unpleasant and emotional experience associated with actual or potential tissue damage or described in terms of such damage’ [2]. Chronic pain is pain that persists for more than 3 months in an individual [3]. Pain is highly prevalent in the Hong Kong population. Studies have revealed that the prevalence of chronic pain ranges from 34.9% to 87.4%, and is high in people aged 18–64 years [4–6]. For the working population in particular, the prevalence of pain is 71.6% [4]. Common pain sites included the neck, bilateral shoulders, back, and extremities [4–6]. Mild to moderate pain intensity has been reported [4, 6]. The pain situation in the Hong Kong population is more serious than that in western countries. The prevalence of pain is reportedly 19% in the United States and 24.9% in Germany with mild intensity [7, 8], levels that are far below that in Hong Kong.

Pain can bring physical discomfort to sufferers and affect them psychologically. Depressive mood and anxiety have been found to be associated with the presence of pain [9, 10]. Patients with chronic pain develop and experience significant levels of depression and anxiety. Stress levels are also related to the presence of chronic pain [11–13]. Psychological stress and stress factors are associated with chronic non-specific neck–arm pain. Pain duration also influences a person’s stress level [12]. According to a longitudinal study, people who suffer from musculoskeletal pain and stress at the same time exhibit poorer working performance and ability [11]. In addition, chronic pain contributes negatively to the quality of life of patients. Surveys have revealed that chronic pain patients with multiple pain sites and pain-related difficulty in engaging in activities are likely to have a lower quality of life [14, 15]. Chronic pain patients have a lower quality of life than those who suffer from acute pain or no pain [16]. One survey focusing on the pain situation of the Hong Kong Chinese population showed that people with higher pain intensities exhibited higher levels of depression and anxiety and a lower quality of life [6].

People often use self-initiated treatments to manage their pain, including taking analgesics, exercising, and resting [17]. Instead of visiting accident, emergency, or outpatient departments, people either self-initiate treatments or use over-the-counter medications to treat their pain [5, 17]. Some even apply multiple treatments such as physiotherapy and traditional Chinese medicine [17, 18]. In addition, they perceive good effects from their self-initiated treatment [17]. Pain services are scarce in Hong Kong. Patients stay in their communities and see general practitioners or primary specialists before visiting pain specialists [18]. Therefore, to manage their pain and especially to meet their preference for pain self-management, it is essential to establish alternative means of targeting the working population. Promoting and enhancing self-efficacy in pain management may be the way to equip the working population with the knowledge and skills to manage their pain.

Self-efficacy is an important measure for determining whether patients can manage their health conditions [19]. Bandura’s self-efficacy theory of behavioural change is a classical model used to explain people’s perceptions and ability to actively participate in activities [20]. Self-efficacy is ‘people’s domain-specific perceptions of their ability to perform the actions necessary to achieve desired outcomes’ [21]. The self-efficacy theory of behavioural change explains how people’s beliefs and expectations contribute to their behaviour. People have their expectations when they engage in activities. When they engage in behaviours, they expect certain outcomes [20]. This leads them to actively participate in activities to achieve expected outcomes. Health promotion and disease prevention can be integrated with the self-efficacy theory of behavioural change [22]. Improving self-efficacy can modulate people’s health behaviour, and the maintenance of health behaviour can be promoted when people gain better control over their health problems. Health studies adopting the self-efficacy theory of behavioural change have demonstrated improvements in self-efficacy when managing chronic illnesses such as osteoarthritis, symptoms of heart failure, and obesity [23–25].

Therefore, in this study the self-efficacy theory of behavioural change will be adopted as the conceptual framework, taking advantage of eHealth, and an electronic pain management programme (ePain) will be designed as the intervention. eHealth is ‘the use of information and communication technologies for health’ [26]. The benefits of using eHealth include an increase in efficiency; enhancements in quality; patient empowerment, encouragement, and education; the exchange of information and communication; and overcoming of the barriers to obtaining health services [27, 28]. With the advancement and advantages of eHealth, people can obtain health education information from the Internet without the limitations of geography and time [29]. Web-based programmes have shown success in enhancing the disease knowledge and self-management abilities of patients with type 2 diabetes, fibromyalgia, and chronic pain [30–32]. There is currently no electronic pain management system that has been tailor-made for the working population and that is in the Chinese language. An electronic pain management programme can meet the demands and service needs of the working population with chronic pain, especially when the population is well equipped with the knowledge to access the Internet and use electronic devices.

### Objectives

This study will aim to develop and evaluate the effectiveness of ePain in improving self-efficacy; reducing pain intensity; decreasing levels of depression, anxiety, and stress; and improving the quality of life of adults seeking to manage chronic pain.

## Methods: participants, interventions, and outcomes

### Trial design and study setting

A two-group, randomized controlled trial will be conducted. ePain will be implemented online to help chronic pain participants self-manage their pain. The participants will be randomized into intervention or control groups. The study protocol will follow the Standard Protocol Items: Recommendations for Interventional Trials (SPIRIT) Statement.

### Preparatory phase

An extensive literature search on pain management methods will be conducted. An online survey will be carried out to collect the preferences and views of the working population on the use of electronic pain management materials. The contents of ePain will be generated by a combination of the literature search and the results of the online survey. The contents will be validated by an expert panel comprising a doctor, a pain nurse, a physiotherapist, and an occupational therapist. Five chronic pain participants who are members of the working population will be recruited to conduct a usability test. Their understanding of the contents of ePain will be examined and their feedback on it will be collected. Their comments and suggestions will be used to improve ePain. The participants will attend a briefing session to understand the objectives of the study, and the applications and functions of ePain. The participants will access ePain with the testing account and password. They will then complete the Computer System Usability Questionnaire to provide information on their user experience and overall evaluation of ePain [33].

### Interventions

#### Conceptual framework

This study will adopt the self-efficacy theory of behavioural change as its conceptual framework. Participants joining ePain will expect to improve their pain knowledge and pain management techniques. Their expectations of the efficacy of pain management will then increase. Their self-efficacy will continually improve and this will strengthen their resolve to continue participating in ePain. Through their repeated use of ePain, their expectations of the outcome will lead to a decrease in the intensity of their pain; reduce their levels of depression, anxiety, and stress; and improve their quality of life.

#### Contents of ePain

The participants will register accounts using their e-mail addresses. An e-mail address and password will be required for every login. After registration, the participants will finish the baseline assessment. They will be asked to identify the most painful sites in their body. Based on the participants’ choices, ePain will provide a tailor-made pain site response message when they first enter ePain. The locations of the relevant content will be shown and the participants will be encouraged to visit the content that fits their needs.

The contents of ePain will focus on self-directed learning about pain knowledge and self-management techniques. Three sessions will cover the self-directed learning of pain knowledge and four sessions will cover self-management techniques. These will include ‘Introduction to pain’, ‘Pain related diseases and syndromes’, ‘Occupational diseases related to pain’, ‘How to manage your pain’, ‘Pharmacological approaches to pain management’, ‘The non-pharmacological management of pain’, and ‘Exercise for relieving pain’.

In the sessions on self-directed learning about pain knowledge, the participants will learn the definition of pain, pain theories, the factors contributing to pain, and how pain contributes to an individual’s physicality and psychology. Diseases, syndromes, and occupational diseases in which pain is presented will also be included.

In the session on self-management techniques, evidence-based pharmacological and non-pharmacological interventions will be introduced. The participants will learn about the World Health Organization’s Pain Relief Ladder [34]. Commonly used analgesics such as paracetamol and tramadol will be included. The use of medications, including dosages, side effects, and precautions, will be discussed. Non-pharmacological interventions for pain management such as aromatherapy, massage, and deep breathing exercises will be introduced [35–38]. The participants will be able to view photographs and video demonstrations of pain relief and prevention exercises in ePain. Pain management will also be introduced from the perspective of Chinese medicine. In addition, the participants will learn how to plan their own pain management interventions.

The participants will click the option ‘I understand the contents of the chapter’ to proceed with the quiz. A quiz will be administered at the end of each chapter to ensure that the participants read ePain and understand the chapter. The questions will be generated from the contents of the chapter. The expert panel will review the questions and the chronic pain participants recruited in the preparatory phase will try out the quizzes and provide feedback. Six true/false questions will cover the contents in each chapter. The participants will complete the quiz and the results will be displayed immediately and recorded in ePain. Those participants who had low marks will be contacted through e-mail to remind them to read the contents again.

The ePain platform will provide a portal for the participants to record their experiences and learning on the management of their pain. The use of pharmacological and non-pharmacological methods of pain management will be recorded on a checklist, which will include the type of intervention, the frequency of usage per day and per week, and the perceived effectiveness of the intervention. Individualized records will be saved in ePain, and the participants will be able to retrieve the records.

ePain will be designed in an interactive, colourful, and user-friendly way. The contents will be easy to read. ePain will be presented in traditional Chinese characters.

ePain will offer a message column for the participants to contact the researcher when they are in doubt and provide comments on the use of ePain. E-mails will be sent at weeks 3, 6, and 12 to prompt the participants to complete the assessments. The participants will receive e-mails in subsequent weeks as a weekly reminder to finish the assessments. A function that sends the ePain link to others will be provided to invite new participants.

### Eligibility criteria

The criteria for inclusion in this study are adults aged 15–65 years, with 65 years being the normal retirement age for civilian officers [39]. The participants will have held a formal job or worked for pay or for profit during the 7 days prior to the study [1]. They will be able to read and understand traditional Chinese characters. They will have had non-cancer chronic pain for at least 3 months and scored 1 or higher on a 10-point numeric pain-intensity rating scale in the Chinese version of the Brief Pain Inventory (BPI-C). They will need to own a computer and a mobile phone, and be able to use the devices to access ePain.

### Recruitment

Participants will be recruited through social network webpages, e-mails sent to companies and workers’ organizations, and posters at the university. Information about the study, including the objectives, procedure, duration, and website of ePain, will be shown in the recruitment materials.

### Randomization

The study will use permuted block randomization. A block size of four and an allocation of 1:1 will be adopted. The sequences that have been generated are AABB, ABAB, ABBA, BAAB, BABA, and BBAA, with A as the intervention group and B as the control group. The block sequence will be programmed to ePain and run randomly. The research team cannot modify the sequence and randomization of the sequence in ePain. The team will be blinded to the sequence used to allocate the participants. When the participants register for ePain, they will be assigned at random according to the sequence, and blinded to the sequence.

### Intervention group

The participants in the intervention group will enter ePain after completing the baseline assessment and randomization at week 1. They will be able to access all of the contents of ePain throughout the study period. They will complete the process evaluation at week 3, the post assessment at week 6, and the follow-up assessment at week 12.

### Control group

The control group participants will be able to download a pain education pamphlet after completing the baseline assessment. The pain pamphlet will contain information from the session ‘Introduction to pain’, which will discuss the definition of pain, pain theories, and factors contributing to pain. They will also need to complete the post assessment at week 6 and the follow-up assessment at week 12. ePain will be open to the participants after they complete the follow-up assessment.

### Outcomes

#### Primary outcome

The primary outcome is pain self-efficacy, which will be measured using the Pain Self-Efficacy Questionnaire at weeks 1, 3, 6, and 12. The questionnaire will contain 10 items. A 7-point scale will be used for measurement, ranging from 0 (not at all confident) to 6 (completely confident), with higher marks representing stronger self-efficacy beliefs. The Cronbach’s α coefficient of the measure of the internal consistency of the items is 0.92 [40]. The Chinese version of the Pain Self-Efficacy Questionnaire (PSEQ-HK) has a Cronbach’s α coefficient of 0.93. Its test–retest reliability coefficient is 0.75, and the SF-36BP and the PSEQ-HK are significantly correlated, with *r* = 0.402 [41].

#### Secondary outcomes

Secondary outcomes will include the measurements for pain situations, negative emotions, quality of life, and the feedback from the participants. The BPI-C, which reports the pain intensity and interfaces of pain in a patient’s life, will be used to measure the pain situations of the participants [42]. It has been validated for use in chronic pain patients [43]. The BPI-C has four pain severity scales and seven pain interference measurements, rated on a scale from 0 (no pain) to 10 (pain as bad as you can imagine). The Cronbach’s α coefficient for pain severity is 0.894 and that for pain interference is 0.915 [42].

The Depression Anxiety Stress Scale (DASS-21) will be used to assess the participants’ levels of negative emotion, including depression, anxiety, and stress [44]. The DASS-21 includes three subscales for depression, anxiety, and stress, respectively, with seven items in each subscale. The scores can reflect the following five grades of depression, anxiety, and stress: normal, mild, moderate, severe, and very severe. The DASS-21 was found to have satisfactory internal consistency with reliability, with *p* = 0.94; while for the depression scale *p* = 0.87, for the anxiety scale *p* = 0.69, and for the stress scale *p* = 0.89 [45]. A Chinese version of the DASS-21 is available. The φ coefficients between the depression, anxiety, and stress scales in the confirmatory factor analysis were 0.92 for depression–anxiety, 0.94 for anxiety–stress, and 0.91 for depression–stress [46].

The participants’ quality of life will be reported using the Hong Kong Chinese version of the World Health Organization Quality of Life Instruments (WHOQOL-BREF (HK)) [47]. Two questions and four domains (physical health, psychological health, social relationships, and environment) containing a total of 28 items will address overall quality of life and health condition [47, 48]. A 5-point scale is adopted in the WHOQOL-BREF (HK). The Cronbach’s α coefficients for the four domains were found to be satisfactory, with 0.77 for physical health, 0.8 for the psychological health, 0.59 for social relationships, and 0.76 for the environment. The inter-rater reliability ranged from 0.8 to 0.91 for the four domains [47].

Open-ended questions will be used to collect comments from the participants, including comments on user experience, the design of the webpage, the usefulness of ePain, and items for improvement.

### Sample size

The sample size was estimated based on Ruehlman et al. [30], who aimed to examine an Internet-based programme for the self-management of chronic pain in the United States. Their measure of pain intensity was used to calculate the effect size, as their study did not examine self-efficacy. The effect size was calculated to be 0.47. To achieve an effect size of 0.47, a power value of 0.8, and an α value of 5%, 114 participants will be recruited. The drop-out rate has been calculated with reference to Ruehlman et al.’s study examining the efficacy of an online Chronic Pain Management Programme and William et al.’s study evaluating the effects of an Internet-based behavioural self-management programme on fibromyalgia patients [30, 31]. The drop-out rate was 7.5% in Ruehlman et al.’s study and 10% in William et al.’s study [30, 31]. A 30% drop-out rate needs to be considered to obtain an adequate number of participants. Therefore, 148 participants will be recruited, with 74 participants in each group.

### Data collection methods

**Fig. 1:**
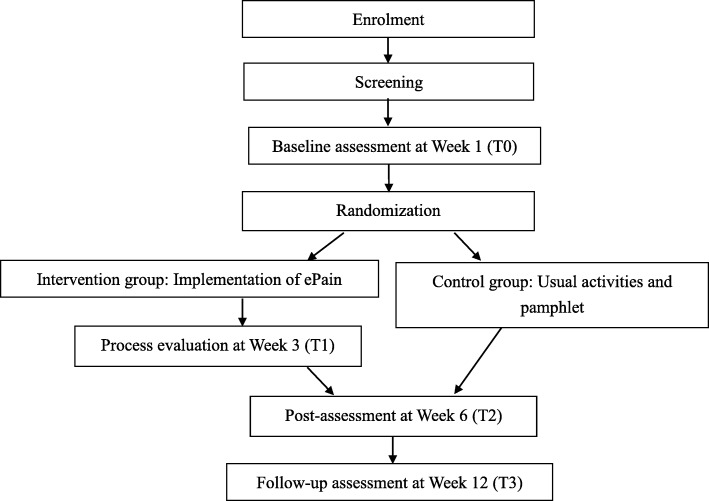
Data collection procedure for the study. ePain electronic pain management programme

As ePain will be an online programme, the data collection procedure will be conducted on the Internet. The participants will use their e-mail addresses to register accounts for individual use. Figure 1 shows the data collection procedure of the study.

**Fig. 2:**
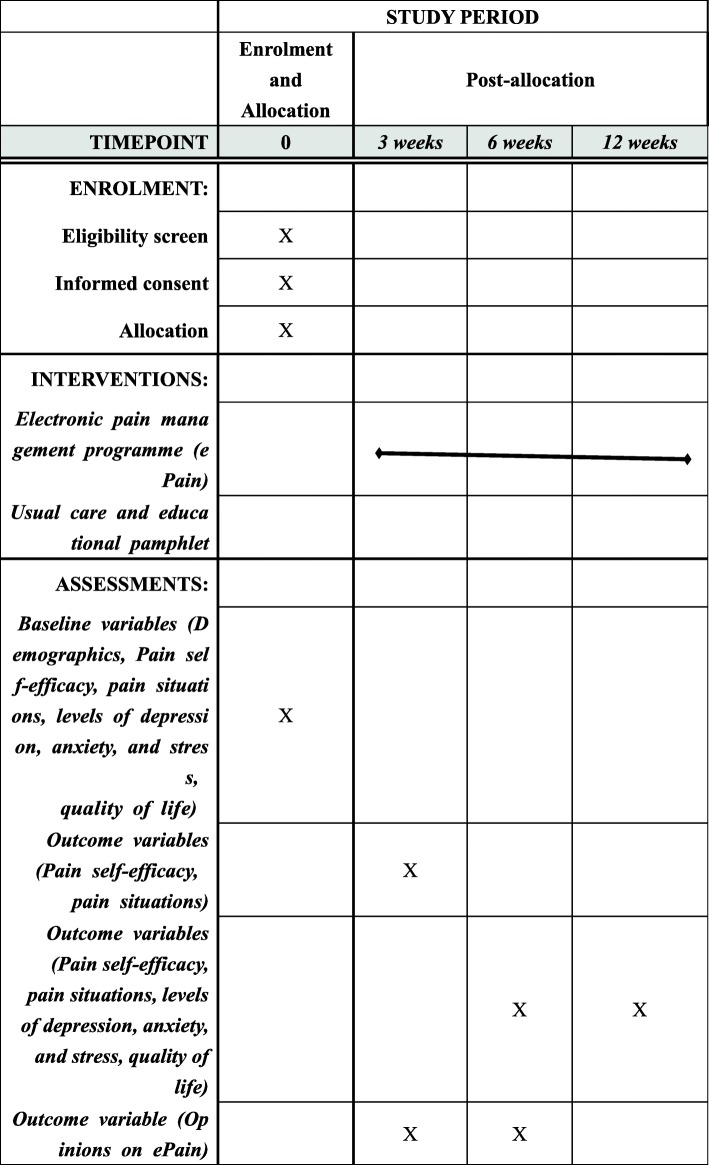
Schedule of enrolment, interventions, and assessments

The outcomes will be measured at different time points. All of the outcomes and demographics will be collected at week 1 as the baseline in both groups. At week 3, the process will be evaluated by collecting information about the participants’ pain self-efficacy, pain situation, experience of using ePain, non-pharmacological interventions for pain management, and comments on ePain in the intervention group. At the week 6 post assessment and the week 12 follow-up assessment, both groups will have to finish the same questionnaire as at baseline. Feedback on ePain will be collected at week 6. To minimize the amount of missing data, messages will appear as prompts when the participants submit their questionnaire, reminding them to complete the missed items. The incomplete items will be highlighted in red to invite the participants to complete them. Figure 2 illustrates the schedule of enrolment, interventions, and assessments.

### Data management

The research team will ensure the confidentiality of the data. The questionnaire and ePain will be hosted on Google, and the collected data will be stored on Google Drive. Google provides information security and encryption services [44]. The data will be retrieved from Google Drive and stored on a computer under password protection. The research team will be the only party able to access the Google account and computer. Passwords will be changed on a regular basis. After the study, all of the data on Google Drive will be downloaded and stored in a password-protected computer. The data will then be deleted from Google Drive.

### Statistical methods

The IBM Statistical Package for the Social Sciences (SPSS) for Windows version 25.0 will be used to statistically analyse the quantitative data. Normality checking will be conducted using the Kolmogorov–Smirnov test. For the descriptive data on the demographics and outcome variables, the means, standard deviations, and percentages will be calculated. For continuous variables, the mean and standard deviations will be presented. The median and a centile range will be reported if the continuous variables do not show a symmetrical distribution.

The data will be analysed on an intention-to-treat basis. Generalized estimating equations will be adopted to investigate the time-averaged difference and differences at each follow-up time. An analysis will be conducted of the within-group effect at week 3, week 6, and week 12, and the interaction between the time effect and the group by group effect (group × time) on the outcome variables, including pain self-efficacy, pain situations, negative emotions, and quality of life. An independent-samples *t* test will be conducted for between-group comparisons at week 3, week 6, and week 12. The significance level will be set at 0.05. Missing data will be analysed using Missing Values in SPSS. The last observation carried forward method will be used to replace the missing data [49, 50].

The Consolidated Standards of Reporting Trials (CONSORT) will be adopted to write up the final report.

## Discussion

Different studies using web-based programmes have demonstrated satisfactory results for their targeted populations [31, 32]. Using web-based programmes to administer health education to the public has become a trend due to the efficiency of this approach in getting in touch with and delivering information to participants [27]. As information technology develops more quickly, both hardware and software will greatly facilitate the spread of information to the public.

This study will adopt the self-efficacy theory of behavioural change to develop an intervention. ePain will provide step-by-step illustrations of pain knowledge and interventions for participants to improve their pain situations. People like to use self-initiated treatments to relieve pain [17]. ePain will provide information about different types of self-initiated treatments, including their correct use. Therefore, this study will use ePain to examine the improvement in the pain self-efficacy of the participants, which previous studies have yet to consider as a primary outcome [30].

The participants in ePain will benefit from improved pain self-efficacy and decreased pain intensity. They will be able to learn and understand pain as they go through the chapters. ePain will introduce various pharmacological and non-pharmacological interventions. The participants will be able to plan and choose their preferred pain relief methods. As psychological distress is associated with pain, the participants will experience lower levels of psychological distress. The anticipated effects of pain management can be sustained; ePain is an electronic pain management programme that will be available on the Internet for the public to access and it will be a cost-effective intervention.

One limitation of the study is the small size of the sample, which could limit the generalizability of the study. The calculation of the effect size is preliminary, based on a previous intervention study on pain intensity. Because there is no information about pain self-efficacy, our estimate of the size of the sample that will be required to determine a significant effect may be an underestimate or an overestimate. However, the optimal sample size can be calculated from the preliminary findings of the study after an accurate estimate is obtained of the standard deviation and the standardized effect size of the primary outcome variable ‘pain self-efficacy’. Another limitation of the study is the selection bias. Participants need to have access to the Internet or to have an electronic device to access the Internet to join the study.

This study will be the first in Hong Kong to examine the effectiveness of an electronic pain management programme that targets the working population. The programme will suit people’s work–life schedules and facilitate how they learn about and plan for pain management. Furthermore, ePain will contain Chinese cultural content that fits the needs of the Chinese population. Indeed, ePain will demonstrate a mix of the East and the West.

The study will attempt to improve the pain self-efficacy; pain intensity; levels of depression, anxiety, and stress; and quality of life of the working population. As an electronic pain management programme, ePain may offer an intervention that improves the pain and psychological status of the working population. Pain education materials can be delivered efficiently through the Internet. The contents of ePain will be tailor-made for the pain situations of the working population and be suitable for pain education. The results of the study will demonstrate how e-learning can help to improve the pain situations of the working population.

### Trial status

Protocol version 1 (23 October 2018). Recruitment began on 1 November 2018. Participant recruitment was completed on 1 January 2020.

This manuscript is re-submitted as recommended by the editor. The initial submission was in February 2019 (Manuscript ID: TRLS-D-19-00183) when the recruitment of participants was in progress.

The approximate date of completion is 1 April 2022.

## Supplementary information

**Additional file 1.** SPIRIT 2013 Checklist: Recommended items to address in a clinical trial protocol and related documents.

## Data Availability

The datasets used and/or analysed during this study are available from the corresponding author on reasonable request.

## Abbreviations

BPI-C: Chinese version of the Brief Pain Inventory
CONSORT: Consolidated Standards of Reporting Trials
DASS-21: Depression Anxiety Stress Scale
ePain: Electronic pain management programme
PSEQ-HK: Chinese version of the Pain Self-Efficacy Questionnaire
SPIRIT: Standard Protocol Items: Recommendations for Interventional Trials
WHOQOL-BREF (HK): Hong Kong Chinese version of the World Health Organization Quality of Life Instruments
